# Obesity as a Perceived Social Signal

**DOI:** 10.1371/journal.pone.0003187

**Published:** 2008-09-11

**Authors:** Manasee Mankar, Radhika S. Joshi, Prajakta V. Belsare, Maithili M. Jog, Milind G. Watve

**Affiliations:** 1 Department of Microbiology, Abasaheb Garware College, Pune, India; 2 Department of Biotechnology, Abasaheb Garware College, Pune, India; 3 Anujeeva Biosciences Pvt Ltd., Pune, India; National Institute of Child Health and Human Development/National Institutes of Health, United States of America

## Abstract

Fat accumulation has been classically considered as a means of energy storage. Obese people are theorized as metabolically ‘thrifty’, saving energy during times of food abundance. However, recent research has highlighted many neuro-behavioral and social aspects of obesity, with a suggestion that obesity, abdominal obesity in particular, may have evolved as a social signal. We tested here whether body proportions, and abdominal obesity in particular, are perceived as signals revealing personality traits. Faceless drawings of three male body forms namely lean, muscular and feminine, each with and without abdominal obesity were shown in a randomized order to a group of 222 respondents. A list of 30 different adjectives or short descriptions of personality traits was given to each respondent and they were asked to allocate the most appropriate figure to each of them independently. The traits included those directly related to physique, those related to nature, attitude and moral character and also those related to social status. For 29 out of the 30 adjectives people consistently attributed specific body forms. Based on common choices, the 30 traits could be clustered into distinct ‘personalities’ which were strongly associated with particular body forms. A centrally obese figure was perceived as “*lethargic, greedy, political, money-minded, selfish* and *rich*”. The results show that body proportions are perceived to reflect personality traits and this raises the possibility that in addition to energy storage, social selection may have played some role in shaping the biology of obesity.

## Introduction

Obesity and related disorders are a growing concern throughout the world. Although there are genetic predispositions to obesity, the number of genes involved is very large [1]. Furthermore the rapidly increasing frequency of overweight and obese individuals in many parts of the world cannot be due to increase in frequencies of any of the obesity related genes. Therefore more attention is focused on gene-environment-behavior interactions contributing to the obesity epidemic. Classically obesity is viewed as a means of energy storage. One of the popular classical concepts has been that of metabolic thriftiness [2], [3]. The thrifty gene is said to confer the ability to store fat at times of food abundance and allow reutilization during food crunch. The thrifty phenotype [4] or thrifty epigenotype [5] concepts differ from thrifty gene concept in that they assume developmental plasticity and fetal programming rather than a set of genes to be responsible for thriftiness. Despite the differences between thrifty gene and thrifty phenotype hypotheses, the central axiom of thriftiness has remained largely unchallenged. These hypotheses have not been rigorously tested and many serious doubts about its validity have been raised [6]–[8]. The current perception is that obesity represents a positive energy balance, i.e. energy intake consistently exceeds energy expenditure and whether the current obesity epidemic is because of increased energy intake or reduced energy expenditure or both is seriously debated. Energy expenditure measurements using doubly labeled water have revealed that there is little difference in the total daily energy expenditure across societies, across lifestyles and over time [9], [10]. This implies that energy intake might have increased. It is not clear however whether increased availability of food is the only factor responsible for increased food intake or other psycho-social factors are involved.

It is becoming increasingly clear, on the other hand, that adipose tissue has many more active roles than being an energy storage tissue alone. Adipose tissues are active endocrine organs directly or indirectly affecting metabolism, immunity, sex, reproduction as well as cognitive brain function [7], [11]–[14]. Many social and behavioral angles of obesity are also coming to light recently. O'Rahilly and Farooqi [15], reviewing all known genetic mechanisms of obesity, concluded that obesity is a more of a neuro-behavioral than a metabolic phenomenon. Christakis and Fowler [16] demonstrated that obesity spreads through social networks. The mechanism behind the apparently socially contagious nature of obesity is not yet known. Christakis and Fowler [16] speculated on psychosocial means, such as changing norms about the acceptability of being overweight. An alternative possible reason can be speculated based on Briers *et al* [17] concept of a cross talk between food and money. Money is a very recent phenomenon in the evolutionary history of humans and therefore separate brain centres to handle money related emotions and information processing are unlikely to have evolved. The brain areas involved in handling food related emotions and information were presumably exapted to handle money. Therefore there could be a cross talk between the neural mechanisms of handling money and food. It's known that the region of the orbitofrontal cortex involved in processing food rewards is also involved in processing money rewards [18], [19]. Briers *et al* [17] showed that hunger affects money related decisions and the desire for money increases hunger. It is also possible that the desire to accumulate wealth results into a tendency to store fat [7]. Money and status related attitudes can be culturally transmitted through social networks and this may partly explain the network epidemiology of obesity.

There are further suggestions that obesity may act as a social signal [7], [20] revealing the past and present resourcefulness of a person. The role of central obesity is of particular interest. It is well known that central obesity is a better predictor of obesity related disorders and that visceral adipose tissue is metabolically more active than subcutaneous fat [21]. If the metabolic and behavioral role of adipose tissue and fat distribution as a social signal co-evolved, it makes much sense that metabolically active fat should be deposited abdominally. Subcutaneous fat is difficult to differentiate from muscle mass and therefore can be of little signal value. Abdominal fat, on the other hand, changes the body proportions substantially and therefore stands out quickly. For a person approaching from a distance, body proportions can be perceived much before facial expressions. Further the theory of honest signaling or the handicap principle states that only costly signals can be evolutionary-stable honest signals [22]–[24]. Fat has a high energy cost and therefore signaling by fat can evolve to be honest.

People are known to make personality judgments very quickly based on facial characters. The judgments are made instantaneously and thinking for a longer time appears to make little difference [25], [26]. Furthermore subjects may not be able to state how they made these judgments suggesting that the judgments are not always made at a conscious level. Most of the studies are restricted to facial features and whether other parts of the body are used by people for instantaneous personality judgments has not been studied.

The hypothesis that fat has a social signal value can be tested at various levels. We test here only one aspect of it that is whether body proportions, as influenced by fat, are perceived to reflect any traits related to the physique, nature, attitude, moral character and status of a person; opening up a number of new questions such as whether the perception is right, whether the signals are honest and whether the associations are culture specific or universal.

## Methods

**Figure 1:**
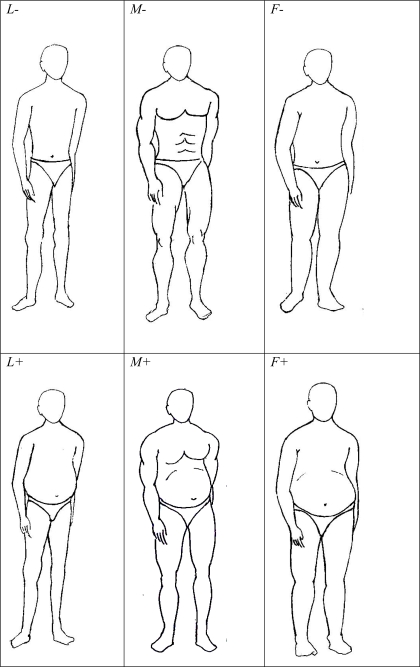
The six body forms used in the study: Three types of body forms were as shown in columns. 1. Lean (*L* - narrow shoulders, thin torso and extremities, knee and elbow joints thicker than thy and arm diameter). 2. Muscular (*M* – Broad shoulders, curved extremities, chest and abdominal muscles shown, thy and arm diameters greater than knee and elbow joints). 3. Slightly fat and feminine (*F* – rounded shoulders, cylindrical extremities). Each of the three body forms was represented with (designated by +) and without (−) abdominal obesity as shown in rows. The sequence of these figures was randomized during the test and the figures were labeled serially by alphabets.

Faceless drawings of three male body forms namely lean, muscular and slightly fat and feminine, each with and without abdominal obesity (designated as *L−, L+, M−, M+, F−* and *F+* respectively) [fig 1] were shown in a randomized order to a group of 222 respondents comprising 140 females and 82 males. All respondents were science students of an age group between 18 and 22 who voluntarily participated in the study with an informed consent. A list of 30 different adjectives or short descriptions of personality traits was given to each respondent and they were asked to allocate the most appropriate figure to each of them independently. The respondents were instructed to choose only one figure for each trait and not to leave any trait without a choice. Further they were asked to make a random choice if they did not find any ‘reason’ to assign any of the figures to a given trait and to note whether a given choice was with conscious reasoning (they were not expected to write reasons) or a random or ‘just felt like’ choice. The traits included those directly related to physique [*strong, physically aggressive, lethargic, disease prone, swift, rough and tough*] those related to nature [*brave, friendly, talkative, intelligent, stupid, methodical*], attitude [*confident, conscious about looks, money minded, physical risk avoider, business risk avoider, depressed*], moral character [*greedy, selfish, political, kind, loving, honest*] and also those related to social status [*status conscious, rich, influential, dominating, successful, modern*]. The study was restricted to male body forms for the fear that the social constraints and taboos on displaying naked female body forms in educational institutes may cause reluctance or bias the responses.

### Statistical methods

For testing significance of choice of figure for each of the 30 traits separately, the null hypothesis that subjects choose any figure randomly for a given personality trait was tested using χ^2^ test with df = 5. For separately testing the effects of the three types of body forms and presence or absence of central obesity, frequencies were pooled in three and two appropriate categories respectively for χ^2^ tests. To test whether the effects of body form and abdominal obesity were independent or interacting, χ^2^ test for independence was performed using a three by two contingency table. Whether the gender of the respondent influenced the choice was also tested by a χ^2^ test for independence. Since a large number of tests were being carried out it was possible that some of the tests turned out to be significant by chance. As a possible solution the level of significance was reduced in inverse proportion to the number of tests being performed [27]. Since 5 different χ^2^ tests were being performed on 30 traits the working cut off should have been 0.05/150. We choose to be even more conservative by choosing α = 0.0001.

To test whether the subjects consistently associated some personality traits with each other, similarity scores were given to each pair of traits based on the frequency with which a given subject choose the same figure for both the traits. Statistical significance of pair-wise association was tested based on the following considerations. The null hypothesis that one of the six figures is assigned randomly to a given trait implies that the probability that two traits shared the same figure for a given respondent should be 1/6. The chance similarities were assumed to be binomially distributed and therefore the mean *n.p* (where p, the probability of a match = 1/6 and n = total number of responses for the given traits) was assumed to have an accompanying variance *npq.* Since *n* was large, the distribution could be considered approximately normal and similarity scores greater than *np + 2 s.d.* were considered significant positive associations and those less then *np − 2 s.d.* as significant negative associations. Based on a similarity matrix of all possible pairs of traits, cluster analysis was performed using unweighted pair group average method. Distinct clusters were defined based on the significance level calculated as above. For each of the clusters the proportions of each of the figures that contributed to the total similarity scores of the cluster were represented as a pie chart.

## Results

Analyzing separately for each trait, the null hypothesis of random responses was rejected in all of the 30 traits. Since a large number of statistical test were involved a conservative level of significance was preferred. On using α = 0.0001, only one of the traits lost significance [Table 1]. All others remained highly significant. Body form and abdominal obesity both appeared to influence the choice in an independent manner in majority of the cases. There was significant interaction between the perception of body form and abdominal obesity in only 10 out of the 30 traits.

**Table 1:** Predominant positive and negative associations of personality traits with different body forms:

Trait	Pre-dominant positive association	Pre-dominant negative association	χ ^2^ for random responses	χ ^2^ for body form alone	χ ^2^ for abdominal obesity alone	χ ^2^ for in-dependence	χ ^2^ for sex difference	% respondents assigning with reason
Physically aggressive	M−	L+, F+, L−	536.22 *	259.16 *	104.07 *	14.87	8.67	56.76
Strong	M−	L− , L+, F+	726.65 *	319.16 *	159.21 *	6.73	8.48	65.77
Lethargic	F+	M−, L−	244 *	89.21 *	93.40 *	10.12	24.53	45.95
Disease prone	L−, F+, L+	F−, M−	183.73 *	121.97 *	8.72	62.60 *	27.23 *	57.21
Swift	L−	F+, M+	61.94 *	13	48.72 *	2.04	9.35	32.43
Rough and tough	M−	L+, F+, L−	560.54 *	250.29 *	127.13 *	9.23	6.62	58.11
Confident	M−, F−	F+, L+	208.81 *	37.48 *	133.26 *	12.99	36.30 *	46.40
Conscious about looks	M−	F+, M+, L+	215.73 *	67.81 *	73.80 *	21.14 *	5.02	52.25
Money minded	F+	M−	46.59 *	15.11	23.35 *	10.25	8.01	27.48
Physical risk avoider	L−, F+	M+, F−, L+	50.70 *	2.78	4.05	43.69 *	7.04	49.55
Business risk avoider	F−	M−	21.02	11.05	2.59	11.94	14.22	22.97
Depressed	L+	M−, F−	161.89 *	137.92 *	18.45 *	29.88 *	9.27	38.74
Rich	F+	L− , L+	225.35 *	131.38 *	52.54 *	1.33	17.34	42.34
Influential	M−	F+, L+	98.65 *	30.35 *	50.61 *	7.76	24.14	33.33
Dominating	M−	L+, L−	147.40 *	107.86 *	22.07 *	1.74	12.79	37.84
Successful	F−	L+, F+	171.62 *	50.19 *	83.31 *	10.90	22.37	34.23
Status conscious	M−	L+, L−	48.97 *	34.89 *	11.26	0.22	18.53	30.63
Modern	M−	L+, F+	385.46 *	125.11 *	162.61 *	1.52	10.31	45.05
Brave	M−	L+, F+	294.65 *	103.89 *	142.72 *	4.95	14.34	46.40
Friendly	F−	M−, F+	178.22 *	44.35 *	56.50 *	56.05 *	18.56	33.33
Talkative	L−, F−	M−, F+	68.49 *	14.38	22.07 *	30.04 *	19.34	23.42
Intelligent	F−	F+, M+	189.57 *	39.35 *	106.83 *	19.62 *	22.02	28.38
Stupid	F+, L+	M−, F−	89.94 *	18.92 *	64.86 *	4.51	3.41	20.27
Methodical	F−	F+	91.51 *	10.24	54.50 *	21.28 *	24.33	26.13
Loving	F−	L+, F+, M+	170.38 *	52.73 *	64.86 *	14.36	32.83 *	22.07
Greedy	F+	L−, M−	295.89 *	117.51 *	98.67 *	5.33	7.72	37.84
Selfish	F+	M−, F−, L−	67.03 *	7.24	56.50 *	5.54	15.76	19.37
Honest	F−	F+, M+, M−	154.32 *	38.19 *	64.86 *	23.61 *	30.41 *	27.03
Kind	F−	M−	123.45 *	54.51 *	28.83 *	22.84 *	16.53	25.23
Political	F+	L−, M−	95.84 *	43.97 *	45.04 *	3.49	17.16	31.53

Both body form and abdominal obesity appear to contribute to the judgment. For most of the traits chi square test for independence is non-significant indicating that the contributions of body form and abdominal obesity are independent of each other. Significance level α = 0.0001

Physical characters were associated with the appropriate body forms as expected. The physical traits *strong, rough and tough* and *physically aggressive* were associated with the muscular non-obese [*M−*] figure. *Lethargic* was associated with *F+*. *Disease prone* was significantly associated with *L−* on the one hand and *F+* on the other indicating that people negatively associate both the extremes with health. The trait *swift* was also strongly associated with *L−.* The traits that are not obviously physical were also strongly associated with certain body forms. *Brave, conscious about looks, influential, dominating, status conscious, modern* and *confident* were associated with *M−*; *physical risk avoider, money minded, political, rich, stupid, selfish* and *greedy* were associated most strongly with *F+*; *friendly, intelligent, methodical, business risk avoider, successful, loving, kind,* and *honest* were associated with *F−*; and *L−* was the commonest choice for *swift, physical risk avoider, talkative* and the trait *depressed* was associated with *L+* [table 1].

Gender of the respondent did not influence the choice of figures for 26 out of 30 traits. In the case of the trait *honest,* female respondents voted more for *F− and F+* whereas male respondents preferred *M−* in greater proportion. In the case of three traits namely *disease prone*, *loving* and *confident,* female respondents favored the fat figure and male respondents the lean figure disproportionately more. There were no significant trends for other traits. We also considered the possibility that the BMI of the respondents may influence their choice. For example obese respondents may be more likely to assign ‘good’ traits to obese figures and lean respondents to lean figures. However we did not find any correlation between the respondent BMI and the frequency with which they assigned ‘good’ characters (*brave, confident, friendly, strong, intelligent, kind, methodical, loving, swift, honest, modern, successful*) to obese figures (r = 0.032, p = 0.636).

The respondents were asked whether they could reason out their choices of figures for each trait. For physical traits the proportion of people choosing with reason was significantly higher (one factor ANOVA, df = 4, F = 6.76, p = 0.001) as expected. For traits related to nature, attitude, moral character and social status, there was a high proportion of ‘just felt like’ responses. However the high level of concordance shows that these responses were highly nonrandom. This indicates that most of these choices could have been made at a subconscious level and although respondents largely converged on their choices, they were not able to give explicit reasons.

**Figure 2:**
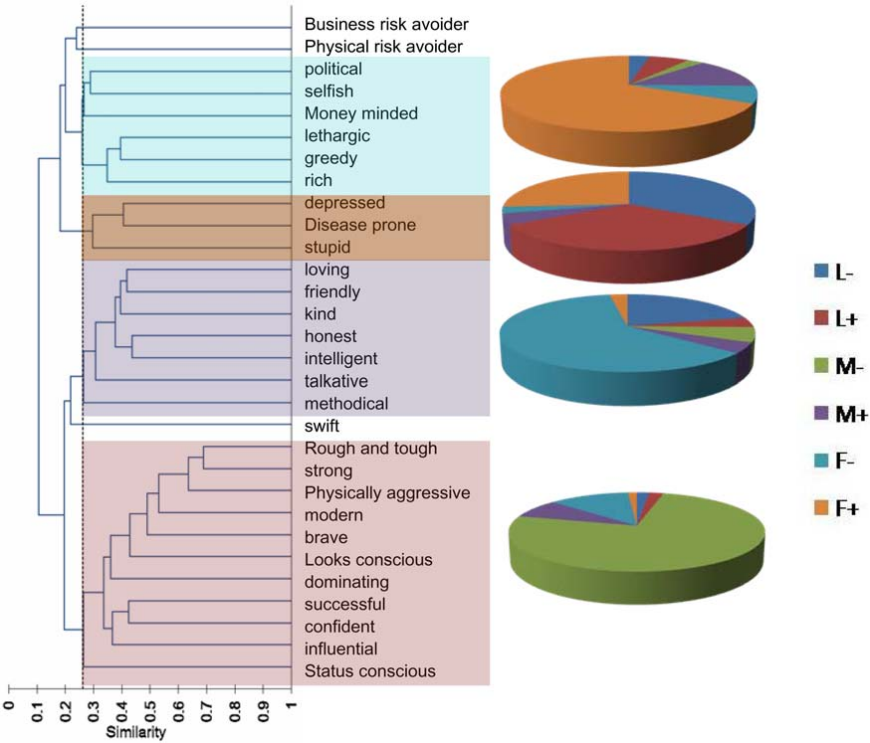
Clustering of personality traits based on the similarity matrix (table 2): Four distinct clusters emerged using the significance level of individual pair (alpha = 0.05) as the cut-off. The four clusters were dominated by different body forms as shown in the pie-charts.

We further asked whether the personality traits had a consistent positive or negative association with each other. The similarity matrix based on common choice of figure by the same respondent revealed that a large number of pair-wise similarities were above the significance threshold for positive association. On the other hand, a large number of pairs showed significantly negative association as well [table 2]. Based on the similarity score the traits were clustered using unweighted pair group average method. At the significance cut off, 4 distinct clusters could be recognized [fig 2]. The first consisted of *strong, rough and tough, physically aggressive, modern, brave, conscious about looks, dominating successful, confident, influential* and *status conscious* and was dominated by *M−*. A second cluster included *honest, intelligent, loving, friendly, kind, talkative* and *methodical* and was dominated by *F−*. A third group of traits comprising *depressed, disease prone* and *stupid* was co-dominated by *L−, L+* and *F+*. The forth distinct cluster consisted of traits including *greedy, lethargic, rich, political, selfish* and *money minded* was dominated by *F+*. The three figures with abdominal obesity namely *F+, M+* and *L+* constituted 87% of this cluster.

**Table 2:** Similarity matrix of all traits based on common choices of figures by respondents: bold face indicates significant positive and underlines negative association

Disease prone	2																											
Selfish	17	33																										
Political	8	**48**	**46**																									
Miser	13	**53**	40	41																								
looks conscious	**109**	9	16	11	14																							
dominating	19	18	**43**	**42**	17	**44**																						
Kind	33	40	16	17	21	22	16																					
Confident	**104**	4	14	13	8	**95**	**50**	**46**																				
Strong	**126**	5	22	13	9	**95**	**64**	18	**72**																			
Physical risk averter	15	**74**	25	32	**51**	18	13	32	6	4																		
straightforward	**61**	19	16	16	17	**49**	37	**47**	**67**	39	15																	
Greedy	7	**67**	**61**	**82**	**46**	6	39	21	5	5	32	23																
methodical	**82**	12	15	15	20	**63**	33	**62**	**77**	**54**	14	64	3															
Detached	15	**60**	40	29	39	17	24	39	14	10	**49**	28	21	23														
Lethargic	9	**84**	**63**	**85**	**50**	21	27	24	5	11	**47**	18	**93**	15	**45**													
Brave	**106**	8	12	10	13	**90**	**53**	33	**104**	**110**	8	**62**	6	**62**	13	7												
business risk avoider	22	38	**42**	31	**44**	19	25	37	22	14	36	30	31	29	40	32	15											
Cunning	24	**44**	37	**46**	**49**	30	35	25	17	16	38	24	**49**	29	30	38	14	28										
Rich	36	**57**	**86**	**78**	**43**	39	**63**	26	32	33	36	26	**82**	30	31	**65**	31	32	**44**									
Friendly	**54**	34	16	20	29	38	26	**75**	**57**	**48**	30	**61**	**62**	**65**	24	28	**45**	35	13	29								
Intelligent	**62**	19	23	13	30	**49**	29	**61**	**74**	37	24	**74**	12	**68**	31	20	**56**	27	24	22	**69**							
Loving	**51**	26	11	23	19	31	29	**82**	**48**	35	18	**51**	24	**54**	32	25	**51**	33	16	37	**78**	**50**						
money minded	23	**57**	**67**	**96**	**48**	18	**52**	22	17	15	40	20	**87**	30	32	**60**	21	37	**58**	**82**	17	20	15					
Bold	**95**	8	18	16	12	**95**	**51**	22	**89**	**92**	11	**52**	16	**54**	18	13	**97**	19	19	29	**47**	**55**	41	20				
Stupid	10	**69**	**50**	**43**	**46**	16	23	24	6	17	**53**	20	**44**	17	40	**62**	9	41	39	**44**	21	7	21	40	6			
Physically aggressive	**86**	21	29	22	19	**73**	**70**	18	**60**	**97**	11	37	22	**48**	24	16	**70**	22	29	**44**	30	27	21	29	**64**	17		
Talkative	31	**46**	35	40	37	**42**	32	**52**	40	16	30	41	35	39	41	38	31	41	35	38	**54**	**49**	39	41	28	21	21	
Status conscious	**69**	29	35	**46**	30	**69**	**60**	26	**62**	**56**	24	45	27	**50**	27	36	**55**	24	33	**58**	41	**43**	37	**52**	**60**	29	**46**	**43**
	physically active	disease prone	Selfish	political	Miser	looks conscious	dominating	kind	confident	strong	Physical risk averter	straightforward	greedy	methodical	detached	lethargic	brave	Business risk avoider	cunning	rich	friendly	intelligent	loving	Money minded	bold	stupid	Physically aggressive	talkative

## Discussion

The results indicate that the respondents significantly agreed on the choice of a particular body form for any given trait. The perceived associations were very strong and taking a highly conservative level of significance did not affect the results. Association of physical traits is not surprising since body proportions directly reflect physique. The physical characters work like positive controls for association and results for all of them are in the expected direction. Of specific interest are non-physical personality characters which show almost equally strong association with some or the other body form. Further traits related to social status, which could be considered independent of the physique by conventional wisdom were also strongly associated. This indicates that body proportions are perceived not only as indicators of health and physique as suggested by Pond [20] but also of nature, attitude, moral character and social status of an individual consistent with the Watve and Yajnik hypothesis [7]. The association of abdominal obesity with *greedy, lethargic, rich, political, selfish* and *money minded* is compatible with the soldier-diplomat dichotomy in metabolism conceptualized by Watve and Yajnik [7], according to whom abdominal obesity is a diplomat trait. Interestingly disease-proneness is associated with two extreme body forms namely *L−* and *F+*. This is also consistent with the metabolic syndrome. Metabolic syndrome is known to be associated with obesity on the one hand and lipoatrophy on the other [28].

The proportion of random versus reasoned choices revealed that the respondents could not answer why and how they associated a particular figure with each of the characters. In an informal feedback after the test many expressed that they were not sure whether whatever they wrote made any sense. Some admitted that they had made completely ‘random’ choices. In spite of these typical reactions from respondents, the responses turned out to be highly non-random. The non-randomness of the responses is also revealed by the clustering of the traits which resulted into consistent personality clusters [fig 2].

The data at present are unable to indicate whether the associations have any biological basis or are shaped by cultural factors such as folklore, literature, cultural norms, role models or any other. A cross-cultural study may reveal whether some of the associations are culture independent universals. We found no effect of responder BMI and only marginal effect of sex. Our sample being small and homogeneous with respect to age, education and cultural background we could not detect the possible effects of these factors. Nevertheless the study demonstrates that people strongly associate body proportions with personality. This raises the possibility that social selection may have been an important force in the evolution of obesity related genes. Since obesity can affect personality judgments, it may influence mate choice- a process that has important direct effects on reproductive success. Other possible effects may include choice of people in possible co-operative alliances and other social interactions that would also ultimately influence reproductive success. It is common in human societies that different stereotyped personalities or role models are associated with different occupations. Even hunter gatherer societies are known to have occupational specializations such as shamans or magic-men. Therefore signaling one's personality by body proportions could have evolved as a natural and effective first level of communication. Similar to primate societies, the first interaction with any individual is likely to be tentative and suspicious. Here the ability to make quick judgments can be highly adaptive. The analysis, although only of a preliminary and exploratory nature, raises a vast array of new questions that should facilitate deeper probe into the co-evolution of psychosocial, physical and metabolic functions.

Identifying the psychosocial aspects of obesity and related disorders has a definite relevance to their control and management. Over the past century the causes of obesity are being increasingly perceived as medical rather than behavioral. As a result the role of individual responsibility in preventing obesity is largely being downplayed [29]. A good understanding of the psychosocial factors can help attaining the right balance in the attitude towards obesity.
